# Comprehensive Analysis of Prognostic Value and Immune Infiltration of Ficolin Family Members in Hepatocellular Carcinoma

**DOI:** 10.3389/fgene.2022.913398

**Published:** 2022-07-19

**Authors:** Liang Sun, Shian Yu, Cairong Dong, Zhengyi Wu, He Huang, Zhendong Chen, Zhipeng Wu, Xiangbao Yin

**Affiliations:** Department of Hepatobiliary Surgery, The Second Affiliated Hospital of Nanchang University, Nanchang, China

**Keywords:** bioinformatics analysis, ficolin family, hepatocellular carcinoma, immunotherapy, prognosis

## Abstract

**Objective:** Ficolin (FCN) family proteins are part of the innate immune system, play a role as recognition molecules in the complement system, and are associated with tumor development. The mechanism of its role in immunotherapy of hepatocellular carcinoma (HCC) is unclear.

**Methods:** In this study, we used the TCGA database, HPA database, Gene Expression Profile Interaction Analysis (GEPIA), Kaplan-Meier plotter, TCGAportal, cBioPortal, GeneMANIA, TIMER, and TISIDB to analyze Ficolin family proteins (FCN1, FCN2 and FCN3, FCNs) in patients with hepatocellular carcinoma for differential expression, prognostic value, genetic alterations, functional enrichment, and immune factor correlation analysis.

**Results:** The expression levels of FCN1/2/3 were significantly reduced in patients with HCC. Among them, FCN3 showed significant correlation with Overall Survival (OS), Progressive Free Survival (PFS) and Relapse Free Survival (RFS) in HCC. FCN1 and FCN3 may be potential prognostic markers for survival in patients with HCC. In addition, the functions of differentially expressed FCNs were mainly related to complement activation, immune response, apoptotic cell clearance and phagocytosis. FCNs were found to be significantly correlated with multiple immune cells and immune factors. Expression of FCN1 and FCN3 differed significantly in the immune and stromal cell component scores of HCC. analysis of the tumor mutation burden (TMB) and microsatellite instability (MSI) of FCNs with pan-cancer showed that FCN3 was significantly correlated with both.

**Conclusions:** Our study provides new insights into the link between the FCN family and immunotherapy for HCC, and FCN3 may serve as a prognostic biomarker for HCC.

## Introduction

Hepatocellular carcinoma (HCC) is one of the most common malignancies worldwide and current treatment mechanisms do not yet lead to a better prognosis (Karimkhani et al., 2017). It is one of the most common malignancies worldwide. Surgical resection, chemotherapy, and intervention have limited effect on the treatment of patients with HCC (Llovet et al., 2018; Zhang et al., 2018). In recent years, immunotherapy regarding HCC has become a research hotspot, and the number of immune drugs associated with HCC has gradually increased. Recent studies have also shown that immunotherapy has a better prospect in HCC (Ghavimi et al., 2020).

Ficolin (FCN) family proteins are present in various tissues, and three Ficolin genes have been identified in humans: FCN1, FCN2 and FCN3. fcn1 is primarily a cellular molecule associated with monocytes and neutrophils; fcn2 and fcn3 are primarily serum molecules. FCNs play a role in innate immunity and environmental homeostasis within tissues. Ficolin is composed of four or more trimers linked together and structurally similar to MBL, and the collagen-like structural domains interact with MBL-associated serine proteases (MAPSPs) to form complexes that activate the lectin complement pathway (Matsushita et al., 2002). Complement activation via lectin is one of the three modes of complement activation (Matsushita and Fujita 2001). In vertebrates and invertebrates, differentiation of self and non-self by lectins is an innate immune strategy (Wotton and Merrill 2007). Previous studies have analyzed the correlation studies between FCNs genes and liver, ovarian, and lung cancers, suggesting a link between FCNs genes and tumorigenesis development (Yang et al., 2016; Jang et al., 2021). In principle, complement-activating proteins not only contribute to cancer development but may also influence the course of the underlying disease.

The mechanism of action of FCNs in tumors is still unclear, and the aim of this study was to analyze the diagnostic and prognostic value of FCNs in hepatocellular carcinoma and the correlation between FCNs and tumor immune mechanisms.

## Materials and Methods

### Expression Levels of Ficolins

The TIMER database (https://timer.cistrome.org/) was used to analyze the differential mRNA expression analysis of FCNs in different human tumors and further analyze the expression analysis and associated prognostic analysis of FCNs in HCC. In addition, We performed immunohistochemical analysis of three pairs of hepatocellular carcinoma and paraneoplastic tissues, which were evaluated in stained sections at ×100 and ×400 magnification, respectively.

### Quantitative Real-Time Polymerase Chain Reaction

In this study, 30 pairs of human HCC tissues and paraneoplastic tissues were collected from the Department of Hepatobiliary Surgery of the Second Affiliated Hospital of Nanchang University, and human hepatocyte cell lines (7,702) and four HCC cell lines (7,721, 97H, LM3 and hu-7) were obtained from the Shanghai Institute of Cell Biology after informed consent was obtained from the patients. All cell lines were cultured in high-sugar DMEM (Solarbio, Beijing, China) supplemented with 10% fetal bovine serum (bio Industries, Beit-Haemek, Israel), 100 µg/ml streptomycin and 100 U/mL penicillin at 37°C in a 5% CO2 humidified incubator. Tissues and cells were extracted for total RNA according to the instructions of Trizol kit (Invitrogen), and cDNA was synthesized using reverse transcription kit (Takara). qRT-PCR was used to detect the expression levels of FCN1, FCN2, and FCN3, and GAPDH was used as an internal control. The primer sequences were: FCN1 Forward primer: CTG​GGC​AGT​CTC​AGT​CGT​G, FCN1 Reverse primer: CCG​ATA​GAA​GTC​CAC​AGA​GCC; FCN2 Forward primer: GGA​ATG​ACA​ACA​TCC​ACG​CC, FCN2 Reverse primer: GGT​TGT​TGT​GGA​ACG​TCA​GG; FCN3 Forward primer: CGT​ACT​TTC​GCC​CAC​TAT​GC, FCN3 Reverse primer: AGT​TGC​TGT​TGC​TTG​AAT​CGT; GAPDH Forward primer:GGAGCGAGATCCCTCCAAAAT, GAPDH Reverse primer: GGC​TGT​TGT​CAT​ACT​TCT​CAT​GG.

### Diagnostic Value and Prognostic Analysis

Predicting the value of FCNs expression levels in the diagnosis of TCGA-LIHC by ROC analysis, Kaplan-Meier plotter (https://kmplot.com/analysis/) , GEPIA and TCGAportal (https://www.tcgaportal.org) were used to assess the correlation between expression of the FCNs family of genes and survival in HCC patients and to calculate HR and *p* values.

### Genomic Data Analysis

CbioPortal (https://www.cbioportal.org) is a comprehensive web resource that presents genomic data from oncology research samples in a visual format. Genomic data types include somatic mutations, DNA copy number alterations (CNAs), mRNA and miRNA expression, DNA methylation, and protein abundance. Genomic data of FCNs were obtained from cBioPortal for visual analysis.

### PPI Network

STRING (https://cn.string-db.org/) is a website for protein interaction. PPI network analysis is conducted to obtain the potential interaction network of FCNs.

### GeneMANIA Prediction Network

GeneMANIA (https://genemania.org/) is used to generate functional analyses of target genes and related genes. Use this site to generate hypotheses about gene function, analyze gene lists, and prioritize genes for functional analysis.

### Correlation Analysis of Ficolins and Immune Infiltration

The TISIDB (https://cis.hku.hk/TISIDB/) database is used to detect interactions between tumors and the immune system. To further elucidate the immune relevance of FCNs genes in cancer, we used the “Immunomodulators” module of the TISIDB database to analyze and assess the correlation between FCNs expression and immunosuppressants, immunostimulants and histocompatibility complexes.

TIMER (https://cistrome.shinyapps.io/timer/) was analyzed mainly for correlation with different immune cell infiltrations and to investigate the link between FCNs and immune cell infiltrations.

### Tumor Microenvironment Analysis

By using the estimate package in R, the algorithm uses gene expression to infer the proportion of stromal and immune cells in tumor samples. Infiltrating stroma and immune cells form a major part of normal cells in tumor tissue and have an important role not only in molecular studies to disrupt tumor signaling but also in cancer biology. ssGSEA algorithm analyzes the association between FCNs and immune cell infiltration in HCC. And the enrichment of 16 immune infiltrating cells in tumor samples was assessed using single sample gene set enrichment analysis.

### Analysis of Tumor Mutation Burden and Microsatellite Instability

Tumor mutation burden and Microsatellite instability are two indicators closely related to Tumor genesis and development. Currently, studies have shown that low tumor mutation load is a poor prognostic factor for tumor patients, and immunotherapy is better for patients with microsatellite instability-high (MSI-H) tumor (Innocenti et al., 2019). By analyzing the correlation between the expression of FCNs and TMB and MSI in 33 kinds of tumors, we discussed the relationship between FCNs activity and HCC mutation. Meanwhile, we further analyzed the association between the expression of FCNs and the score of TMB.

### Analysis of the Expression of Ficolins and the Effect of Immune Checkpoint Inhibitor Therapy

We downloaded immune data of HCC from The Cancer Immunome Atlas (TCIA) database (https://tcia.at/home) to analyze the expression of FCNs with the effect of immune checkpoint inhibitor drug therapy.

### Drug Sensitivity Analysis

CellMiner (https://discover.nci.nih.gov/cellminer/home.do) is a web application developed by the Genomics and Bioinformatics Group at the National Cancer Institute (NCI) to explore drug activity in NCI-60 cell lines. Drugs sensitive to FCNs are explored through analysis of the site’s database.

## Results

### Differential Expression of Ficolins in Hepatocellular Carcinoma Patients

**FIGURE 1:**
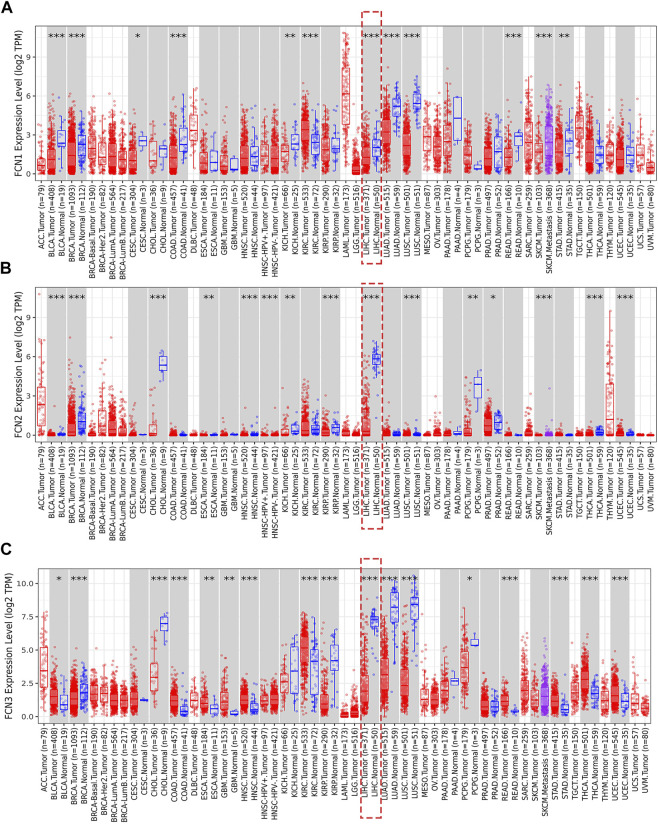
The expression levels of FCNs in different tumor (TIMER). **(A)** FCN1; **(B)** FCN2; **(C)** FCN3.

**FIGURE 2:**
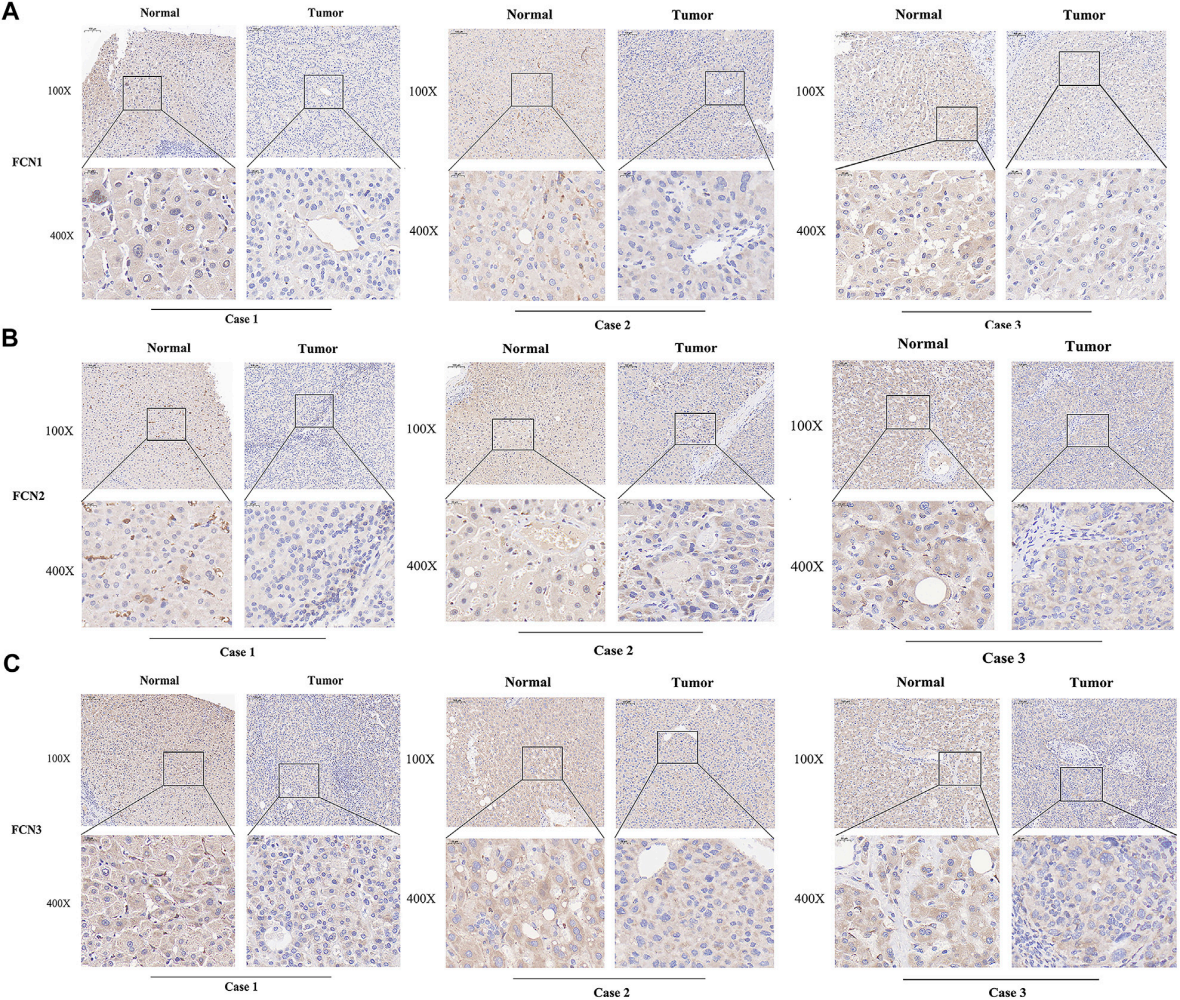
Immunohistochemistry of FCNs in three pairs of HCC tissues. **(A)** FCN1; **(B)** FCN2; **(C)** FCN3.

To explore the differential expression levels of FCNs, we analyzed the expression of FCNs in numerous human tumors using tumor data in the TIMER database, in which the expression of FCNs showed significant differences between tumor samples (*n* = 371) and normal samples (*n* = 50) in TCGA-LIHC (Figures 1A–C). In addition, the IHC results of FCNs showed that FCNs were expressed at higher levels in normal liver tissues. (Figures 2A–C). These results all suggest that FCNs are expressed at low levels in HCC and that they may be a tumor suppressor in HCC.

### Detection of Ficolins Expression in Hepatocellular Carcinoma by q-PCR

**FIGURE 3:**
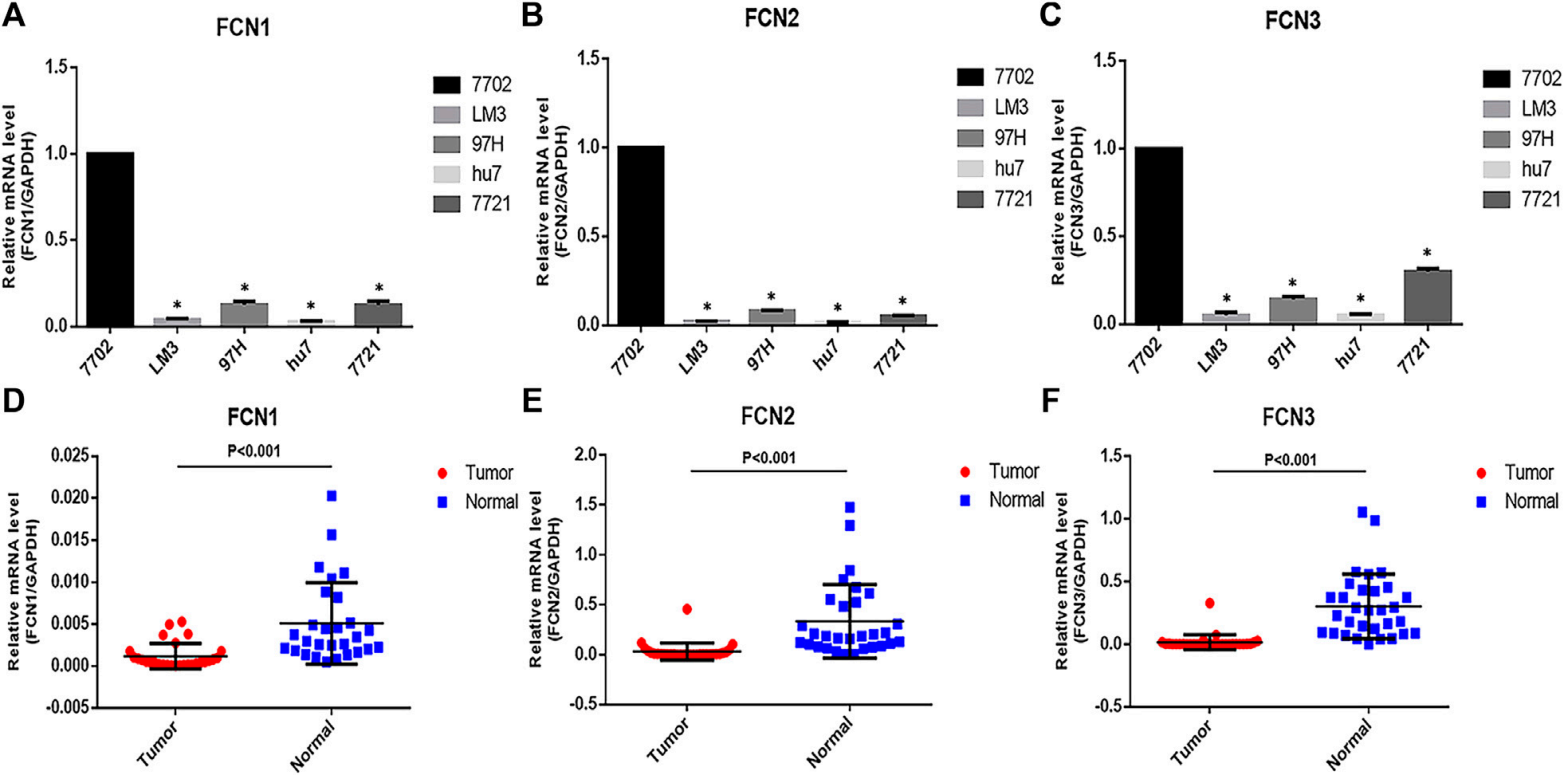
The expressions of FCNswere down-regulated in LIHC cells and tissues. **(A–C)**. The mRNA expression of FCNsin 7,702, LM3, 97H, hu7, and 7,721 cell. **(D–F)**. mRNA expression of FCNs in LIHC tissues. *p < 0.05.

To verify the differential expression of FCNs in HCC, we went to detect the mRNA expression of FCNs in normal hepatocytes (7,702) and hepatoma cells (LM3, 97H, hu7 and 7,721) by q-PCR, and the results showed that the expression of FCNs was significantly lower in hepatoma cells (Figures 3A–C). Meanwhile, we collected 30 pairs of HCC tissues and their paracancerous liver tissues to detect the mRNA expression of FCNs in HCC tissues, and the results showed that the expression of FCNs was significantly lower in HCC tissues compared with paracancerous tissues (*p* < 0.01) (Figures 3D–F).

### The Value of Ficolins in Potential Diagnostic Markers of Hepatocellular Carcinoma

**FIGURE 4:**
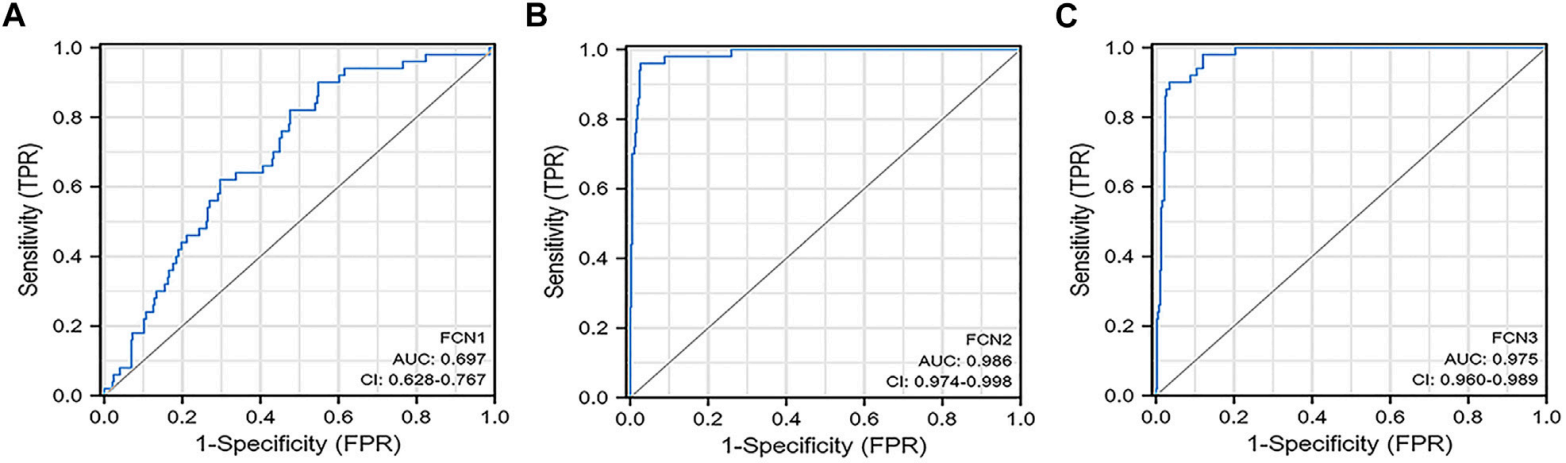
The diagnostic value of FCNs in LIHC patients. **(A)** FCN1; **(B)** FCN2; **(C)** FCN3.

ROC analysis was applied to evaluate the effectiveness of FCNs mRNA expression levels in distinguishing hepatocellular carcinoma from normal liver tissue, and the AUC of FCN1 was 0.697 (95% CI: 0.628-0.767) (Figure 4A) obtained by analysis of TCGA-LIHC data. the AUC of FCN2 was 0.986 (95% CI: 0.974-0.998) (Figure 4B). the AUC of FCN3 was 0.975 (95% CI: 0.960-0.989) (Figure 4C).

### Prognostic Value of the mRNA Expression of Ficolins in Patients With Hepatocellular Carcinoma

**FIGURE 5:**
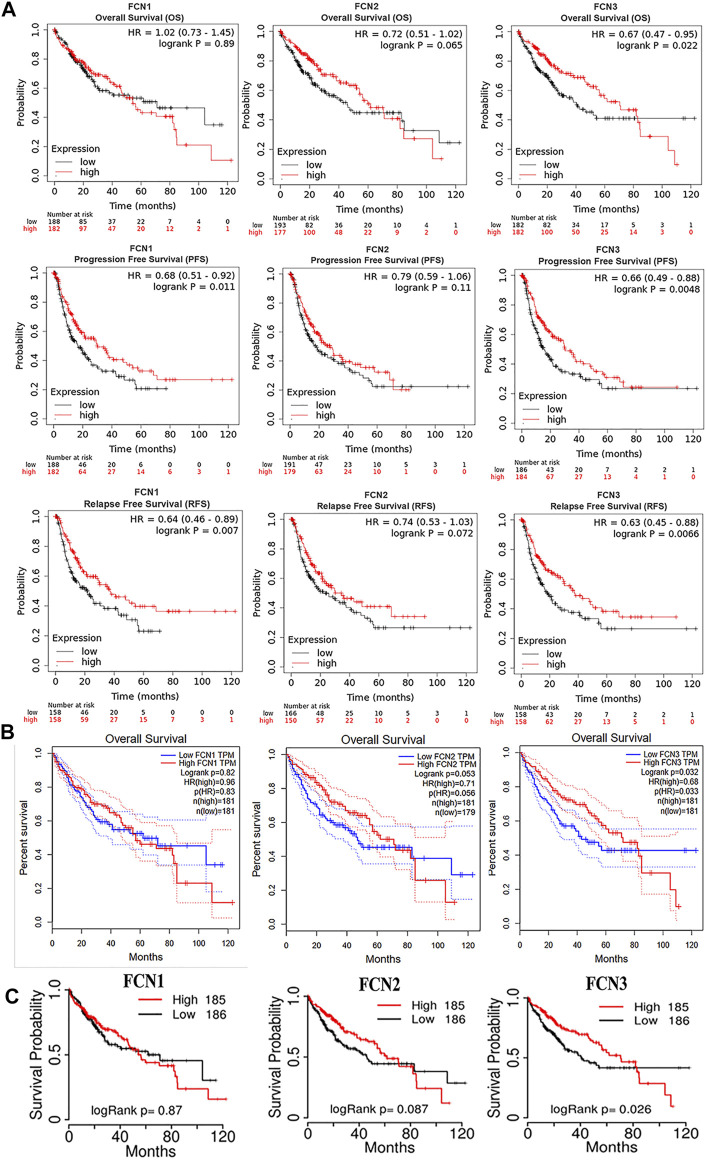
The diagnostic and prognostic value of FCNs in LIHC. **(A)** Kaplan-Meier plotter reveals the overall survival (OS) progression free survival (PFS) and relaps-free survival (RFS) curvesbased on the mRNA levels of FCNs in LIHC patients. **(B)** Overall survival (OS) curves of FCNs in LIHC patients determind using GEPIA. **(C)** Overall survival (OS) curves of FCNs in LIHC patients determind using TCGAportal.

To assess the value of differential expression of FCNs in the progression of HCC, we used a Kaplan-Meier plotter to analyze the overall survival (OS), progression-free survival (PFS), and relapse-free survival (RFS) of FCNs. and the results showed that only FCN3 showed significant correlation with OS in HCC (*p* < 0.05); FCN1 and FCN3 showed significant correlation with PFS and RFS in HCC (*p* < 0.05); while the expression of FCN2 in HCC was significantly correlated with OS, PFS and RFS were not significantly correlated (*p* > 0.05) (Figure 5A).

The correlation between the expression of FCNs and OS of HCC was analyzed using GEPIA and TCGAportal, and it was found that there was a significant correlation between only FCN3 and OS of HCC, and the higher the expression of FCN3, the higher the survival rate of patients (*p* < 0.05) (Figures 5B,C). In addition, we analyzed the association between FCNs and RFS of HCC patients using GEPIA, and found that the higher the expression of FCN1 and FCN3, the higher the survival rate of patients (*p* < 0.05), and there was no significant correlation between the expression of FCN2 and RFS (*p* > 0.05) (Supplementary Figure S1A–C).

The OS results obtained in all three ways suggested a significant association between the expression of FCN3 and the OS of HCC, while for PFS and RFS of HCC, the Kaplan-Meier plotter suggested a significant correlation between the expression of FCN1 and FCN3. The above evidence suggests that FCN3 shows a higher research value in the prognosis of HCC.

### Analysis of Clinical Correlation Between Ficolins and Patients With Hepatocellular Carcinoma

Analysis of TCGA-LIHC data revealed that FCNs were not significantly correlated with Grade stage in HCC patients (*p* > 0.05) (Supplementary Figure S2A). Correlation analysis of FCNs with Stage stage in HCC patients revealed that only FCN3 showed significant correlation with Stage stage (*p* < 0.05) (Supplementary Figure S2B).

### Genetic Alteration, Interaction Analyses and Potential Function of Ficolins in Hepatocellular Carcinoma

**FIGURE 6:**
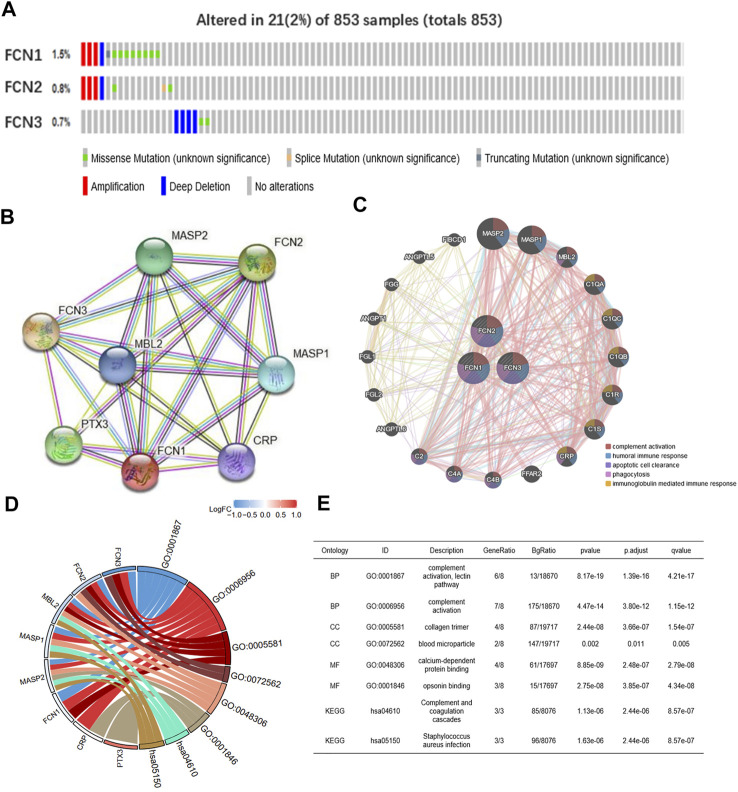
FCNs gene mutation and PPI network constraction. **(A)** Summary of alterations in different expressed FCNs in LIHC. FCNs were altered 21 samples of 853 patients with LIHC, accounting for 2%. **(B,C)** Protein-protein interaction network of different expressed FCNs. **(D,E)** GO and KEGG analysis of the FCNs and correlation genes.

We analyzed the genetic alterations of FCNs in HCC patients using the cBioPortal online tool. FCNs was altered in 21 samples from 853 HCC patients, accounting for 2%. the rates of genetic alterations were 1.5, 0.8 and 0.7% for FCN1, FCN2 and FCN3, respectively (Figure 6A). We performed a protein-protein interaction network (PPI) analysis on FCNs using STRING to explore their potential interactions (Figure 6B). GeneMANIA results showed that the function of differentially expressed FCNs with their neighboring genes (e.g. MASP1, MASP2, MBL2, C1QA, C1R, CRP, and C2, etc.) were mainly related to complement activation, humoral immune response, clearance of apoptotic cells, phagocytosis and immunoglobulin mediated immune response (Figure 6C).

We performed enrichment analysis of FCNs and their associated genes, and GO and KEGG analysis showed that FCNs and their associated genes were associated with complement activation, lectin pathway, collagen trimerization, blood coagulation, calcium-dependent protein binding, conditioner binding, complement system, and *S. aureus* infection (Figures 6D,E).

The gene set variation analysis (GSVA) algorithm was also applied to explore the correlation of FCNs with numerous pathways in HCC. The results showed that FCN1 and FCN3 showed significant correlations (*p* < 0.05) with a large number of pathways in HCC, while FCN2 only showed significant correlations with PPAR_SIGNALING_PATHWAY, CALCIUM_SIGNALING_PATHWAY and ADIPOCYTOKINE_SIGNALING_PATHWAY showed significant correlations (Supplementary Figure S3).

### Immune Cell Infiltration of Ficolins in Patients With Hepatocellular Carcinoma

The above functional enrichment results suggest that FCNs may be involved in the body’s immune response. Previous studies have also shown that FCNs are associated with autoimmune diseases. In the present study, we observed that FCNs were all in a low expression form in HCC; therefore, we hypothesized that FCNs may be involved in regulating tumor immune responses.

**FIGURE 7:**
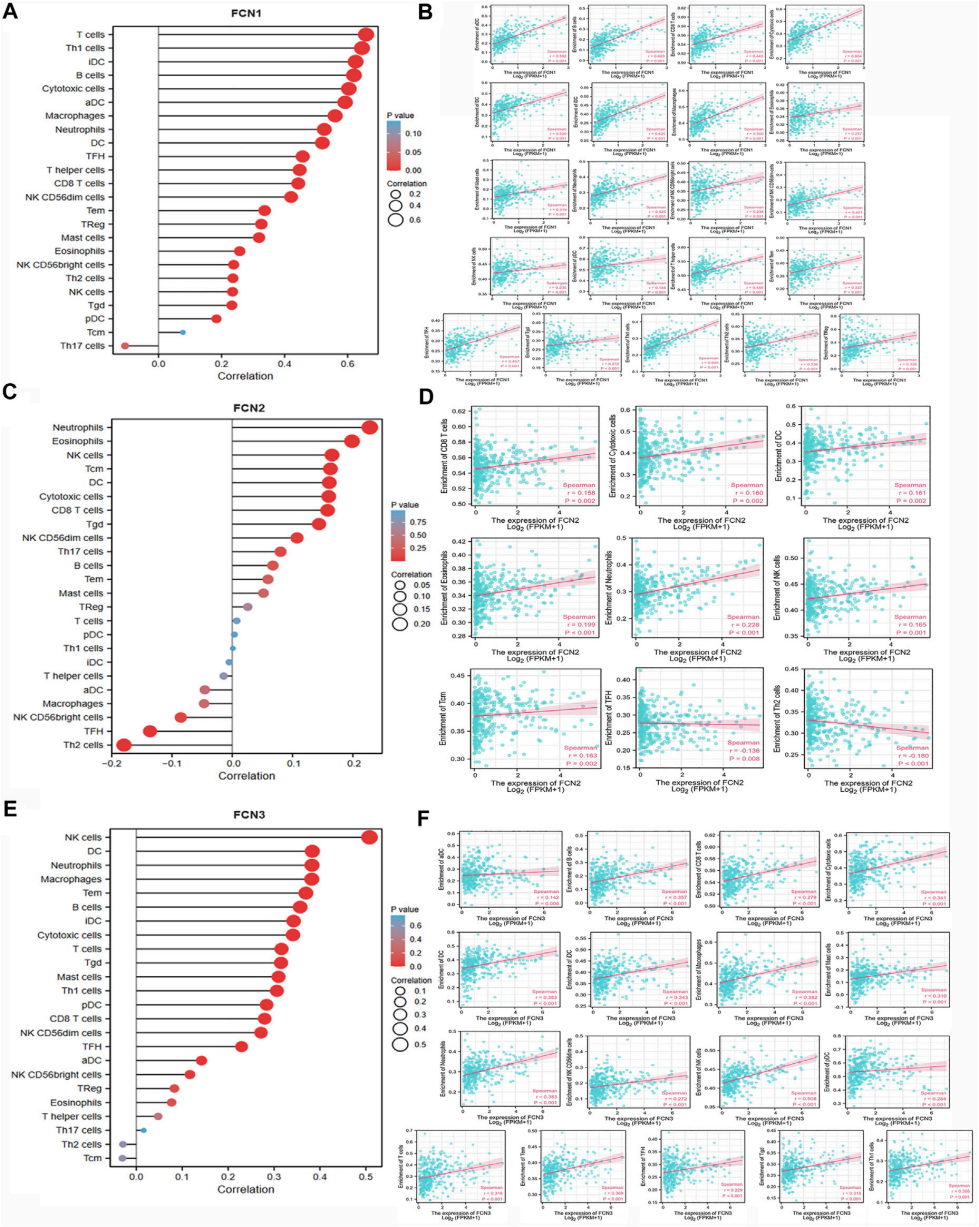
Correlation between FCNs and immune cell infiltration. **(A,B)** FCN1; **(C,D)** FCN2; **(E,F)** FCN3.

The expression level of FCN1 was significantly correlated with the infiltration level of 22 immune cells, and all of them were positively correlated. the expression level of FCN2 was significantly correlated with the infiltration level of 9 immune cells, and it was negatively correlated with TFH and Th2 cells. the expression level of FCN3 was significantly correlated with the infiltration level of 17 immune cells, and all of them were positively correlated (Figure 7) (Supplementary Table S1).

Association between FCNs and immune-related factors and tissue-associated antigens in patients with HCC

**FIGURE 8:**
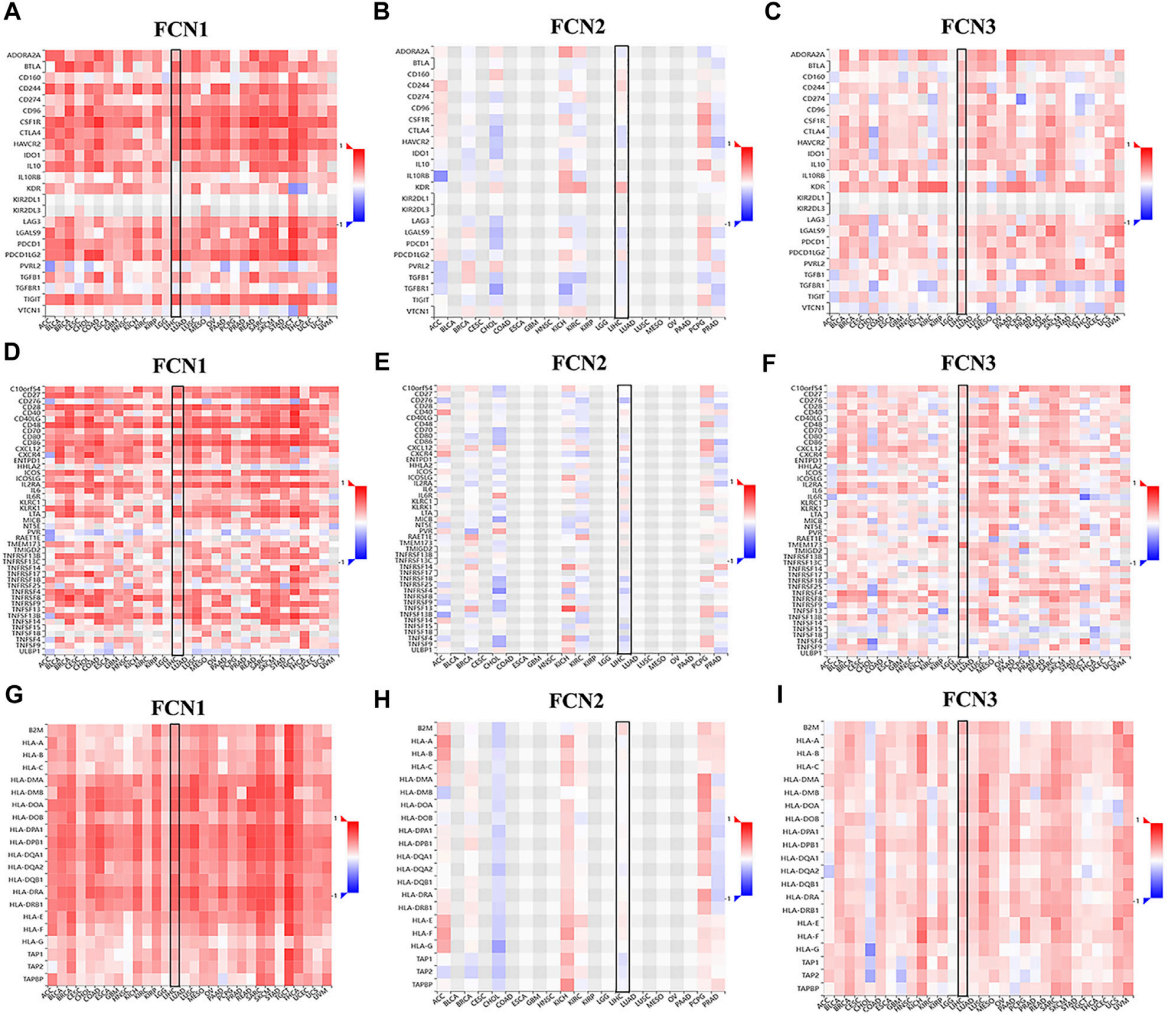
Correlation analysis between FCNs expresion and immunoinhibitors, immunostimulators and majorhistocompatibility complex (MHCs). **(A–C)** Correlation analysis between FCNs expresion and immunoinhibitors **(D–F)** Correlation analysis between FCNs expresion and immunostimulators. **(G–I)** Correlation analysis between FCNs expresion and MHCs.

Immune checkpoint inhibitors (ICIs) are a novel tumor immunotherapy strategy that has gradually improved the prognosis of patients with multiple cancers (Huang et al., 2020). FCNs exhibited correlations with numerous immune cells, and we subsequently analyzed the correlations of FCNs with immunosuppressive agents in different types of human tumors (Figures 8A–C), with immunostimulants (Figures 8D–F), and with histocompatibility complexes (MHCs) using the TISIDB database (Figures 8G–I).

### Analysis of the Tumor Microenvironment of Ficolins in Hepatocellular Carcinoma

**FIGURE 9:**
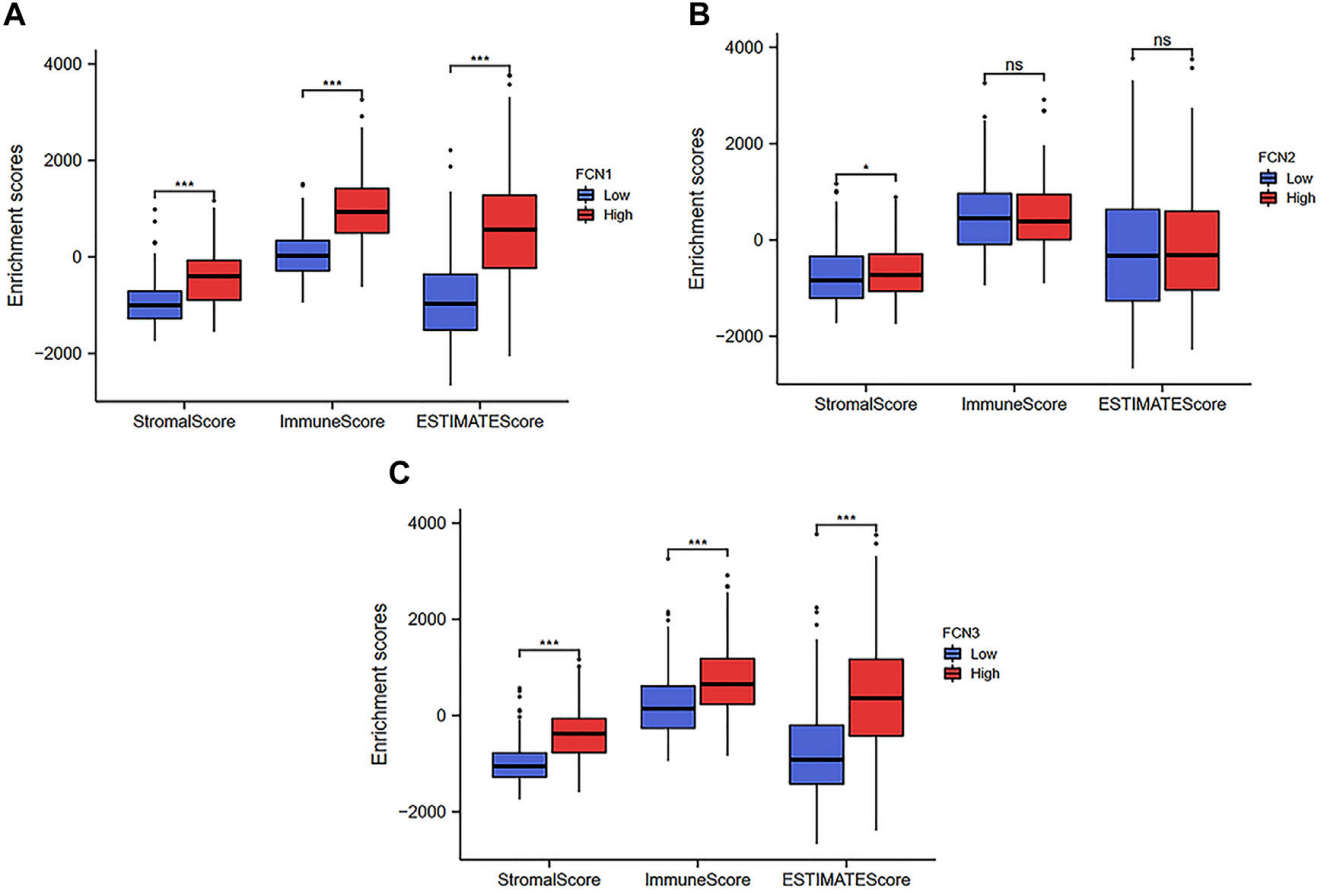
Stromal and immune cell scores in LIHC. **(A)** FCN1; **(B)** FCN2; **(C)** FCN3; *p < 0.05, **p < 0.01, ***p < 0.001.

To explore the role of FCNs in the tumor microenvironment, we used the ESTIMATE package in R language to infer the ratio of immune and stromal cell components by the expression of FCNs to obtain the relationship between the expression of FCNs and the stromal and immune cell scores in HCC. The results showed that the expression of FCN1 and FCN3 showed significant correlation with StromalScore, ImmuneScore and ESTIMATEScore (*p* < 0.001); the expression of FCN2 showed significant correlation with StromalScore only (*p* < 0.05) (Figures 9A–C). We also analyzed the relationship between FCNs and immune infiltration by the ssGSEA algorithm, and the results showed that there was a strong association between FCN1 and FCN3 and numerous immune cells and immune functions, while FCN2 had a relatively poor association with immune cells and immune functions (Supplementary Figure S4A–F).

### Correlation of Ficolins Expression in Pan-Cancer With Tumor Mutation Load and Microsatellite Instability

**FIGURE 10:**
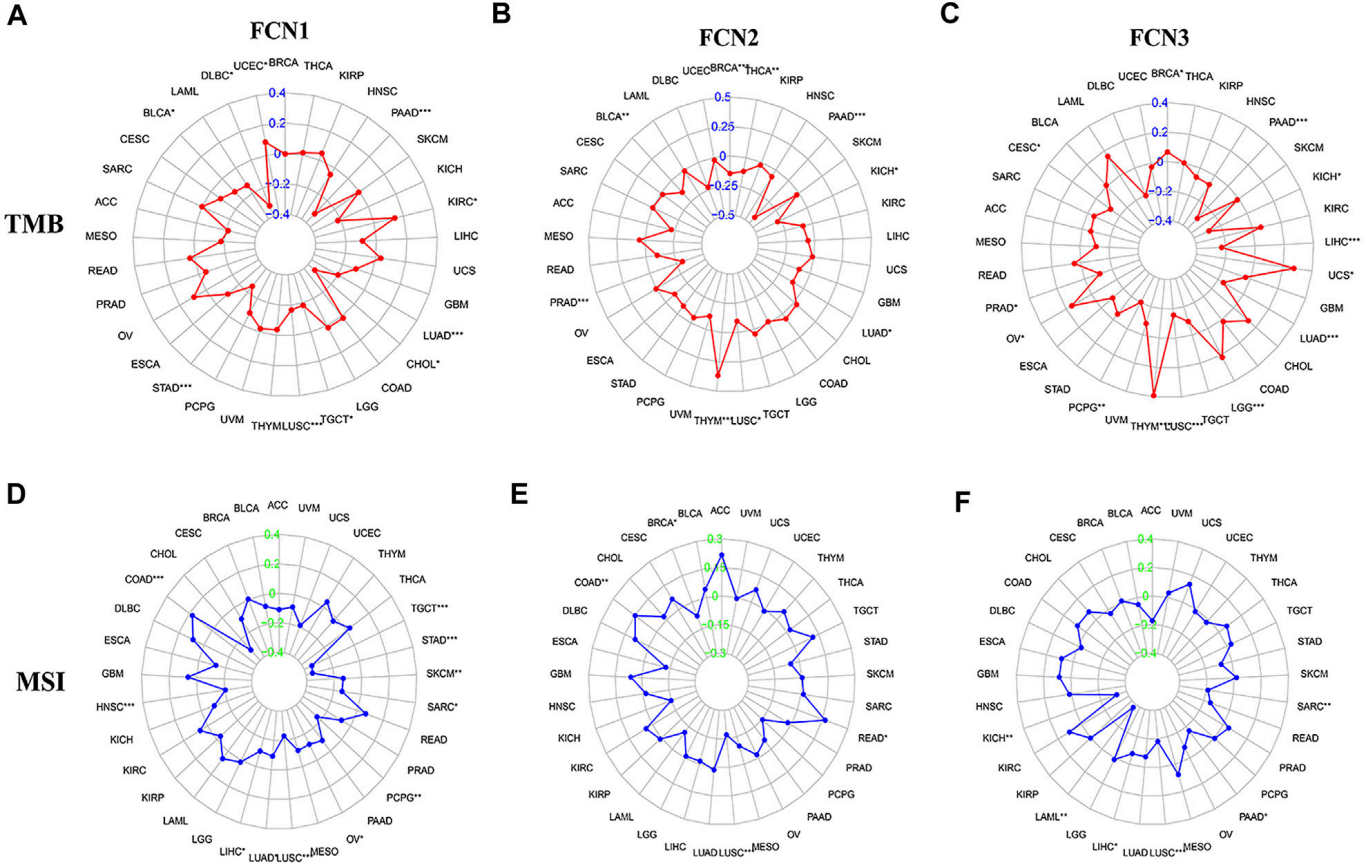
Correlation of FCNs with tumor mutation burden (TMB) and microsatellite instability (MSI) in Pan cancer. **(A,D)** FCN1; **(B,E)** FCN2; **(C,F)** FCN3; *p < 0.05, **p < 0.01, ***p < 0.001.

By analyzing the correlation of FCNs expression with tumor mutation burden and microsatellite instability in 33 type of human tumors, FCN1 expression was found to be significantly correlated with MSI of HCC (*p* < 0.05); FCN3 expression was significantly correlated with TMB and MSI of HCC (*p* < 0.05) (Figure 10). Meanwhile, we further analyzed the association between the expression of FCNs and TMB, and the results showed that FCN3 showed a significant correlation with TMB (*p* < 0.05), and the higher the expression of FCN3, the lower the TMB score. In contrast, there was no significant correlation between FCN1 and FCN2 expressions and TMB in HCC patients (*p* > 0.05). Therefore, FCNs may become immunotherapeutic targets for HCC, with FCN3 having higher research value (Supplementary Figure S5).

### Immune Escape Analysis

**FIGURE 11:**
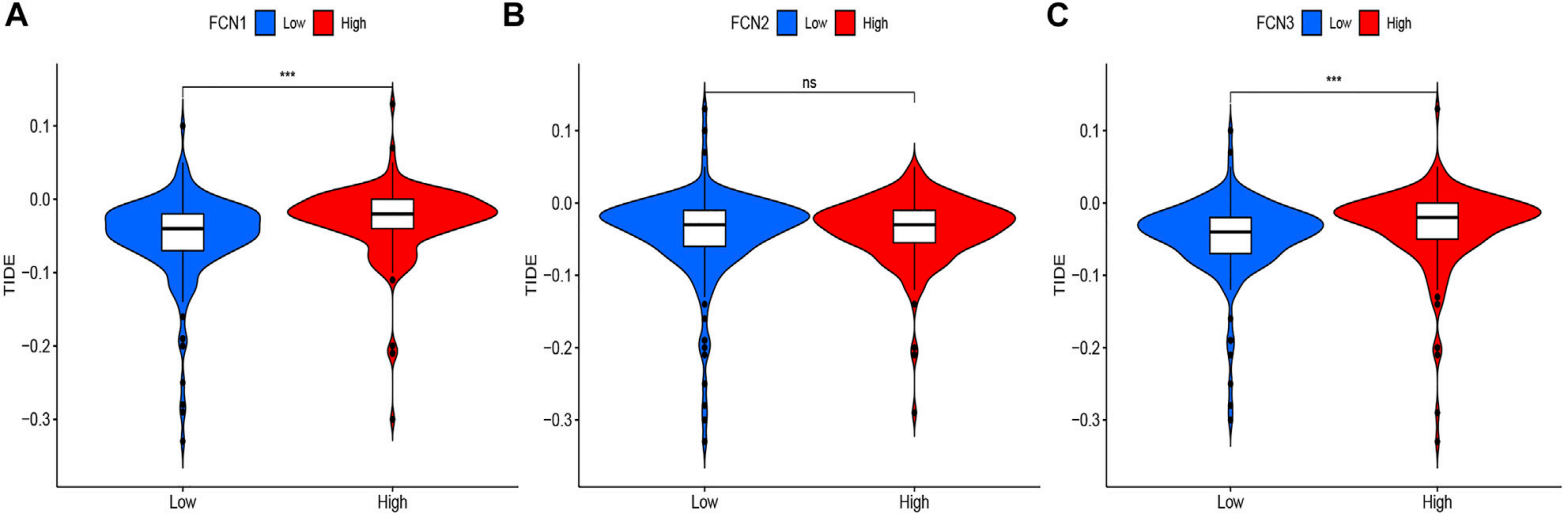
Correlation between FCNs expression and TIDE score. **(A)** FCN1; **(B)** FCN2; **(C)** FCN3; ***p < 0.001.

To further analyze the association between the Ficolin family and immunotherapy efficacy, we analyzed the correlation between the expression of FCNs and immune escape score, and the results showed that the expression of FCN1 and FCN3 were significantly correlated with the TIDE score (*p* < 0.001), and the higher their expression, the higher the risk of immune escape and the worse the immunotherapy efficacy. There was no significant correlation between FCN2 expression and TIDE score (Figure 11).

### Analysis of the Expression of Ficolins and the Efficacy of Immune Checkpoint Inhibitors

analyzed the expression of FCNs with the efficacy of two immune checkpoint inhibitors, CTLA4 and PD-1, and found that the expression of FCN1 and FCN3 was significantly correlated with the efficacy of immune checkpoint inhibitors (*p* < 0.05), and the higher the expression of FCN1 and FCN3, the higher the anti-CTLA4 treatment, anti-PD-1 treatment or both drugs the betters the efficacy. In contrast, the expression of FCN2 was not significantly correlated with the efficacy of anti-CTLA4 treatment, anti-PD-1 treatment and the combination of both drugs (*p* > 0.05) (Supplementary Figure S6A–C).

### Drug Sensitivity Analysis

By analyzing the FCNs with the drugs in the CellMiner database, we show the top 4 drugs with the highest sensitivity to FCNs, respectively. Among them, FCN1 showed significant correlation with Megestrol acetale, Isotretinoin, Imiquimod and Imexon (*p* < 0.05). FCN2 showed significant correlation with Isotretinoin, Imiquimod, Fluphenazine and Oxaliplatin (*p* < 0.05). FCN3 showed significant correlation (*p* < 0.05) with Hydrastinine HCI, Buthionine sulphoximine, Parthenolide and E-7820 (Supplementary Figure S7A–C).

## Discussion

Hepatocellular carcinoma is one of the most common malignant tumors today, and its incidence is on the rise globally (Johnston and Khakoo 2019). The molecular pathogenesis of HCC varies across genotoxicity and etiology, and although our understanding of the pathophysiology and drivers of the disease has improved, this knowledge has not yet been translated into clinical practice. Currently, immune checkpoint inhibitors (ICIs), tyrosine kinase inhibitors (TKIs) and monoclonal antibodies have expanded the therapeutic field for the treatment of HCC (Zongyi and Xiaowu 2020; Feng et al., 2021). Approximately more than half of HCC receive systemic therapy, especially in the advanced stage of HCC. and the field has made significant progress in the development of systemic therapies, with studies reporting significant improvements in overall survival and quality of life for patients (Llovet et al., 2018 ^)^.

Ficolin is a polyprotein consisting of an n-terminal collagen-like domain and a c-terminal fibrinogen-like domain. Its structure is similar to that of mannose-binding lectin (MBL) and complement C1q. Ficolin was first thought to act via the lectin pathway to activate complement (Matsushita et al., 2000). Subsequent studies revealed that Ficolin acts through two distinct pathways: the lectin pathway and a primitive regulatory phagocytosis (Endo et al., 2007). Ficolin has now been shown to act in several species, such as human, bird, mouse, etc. (Fujimori et al., 1998; Sugimoto et al., 1998; Lynch et al., 2005). FCN family genes are closely related to the human innate immune system and, based on their locus and molecular signature, function in the clearing of non-autosomes. FCN1 is expressed primarily in peripheral blood leukocytes and bone marrow, with minor expression in the spleen and lung (Munthe-Fog et al., 2012). FCN2 is a soluble serum protein, that is, thought to play an important role in host innate immunity, is expressed primarily in the liver, and is secreted into the circulation (Le et al., 1998). gnieszka Szala et al. found lower expression of FCN2 in ovarian cancer than in normal ovaries or benign ovarian tumors (Szala et al., 2013). Some studies found that FCN2 as a potential biomarker may have diagnostic value for oral squamous cell carcinoma (Arellano-Garcia et al., 2010). FCN3 is synthesized in the liver by hepatocytes and biliary epithelial cells and secreted into the bile ducts and circulation and is also expressed at lower levels in the heart, kidney, spleen, pancreas and placenta (Akaiwa et al., 1999). Michalskia studies suggest that FCN3 may be involved in the immune response to ovarian cancer and that its expression is associated with the development of ovarian cancer (Michalski et al., 2019). A recent study showed that FCN3 is a tumor suppressor gene that acts as an oncogenic factor in lung adenocarcinoma by inducing endoplasmic reticulum stress (Jang et al., 2021)

The relationship between FCNs and carcinogenesis or their direct interactions with tumor cells has not been extensively studied. In this study, a comprehensive analysis of FCNs was performed in terms of their expression differences, gene mutations, diagnostic value, prognostic value and immune cell infiltration. There are few studies on FCNs in hepatocellular carcinoma, and the present study found that FCNs were significantly less expressed in HCC than in normal liver tissues in terms of expression levels. Moreover, HCC patients with high FCN3 transcript levels were significantly associated with long OS, PFS and RFS. Combined with the present findings, we inferred that FCN3 is an oncogene in HCC that can effectively mitigate the development of HCC and has the potential to be a new target for the treatment of HCC. In this study, we also found by correlation analysis between FCNs and immune infiltration that FCNs were significantly correlated with numerous immune cells and in correlation with immunosuppressants, immune stimulators and tissue-associated antigens, all suggesting a high research value. We also found that the higher expression of FCN1 and FCN3 was associated with a significantly higher score of both immune cells and stromal cells in HCC by analyzing both components. These results reflect the value of FCNs in immunotherapy of HCC.

Previous studies have shown that Ficolin triggers complement activation via the lectin pathway, thereby mediating a range of immune responses, including modulatory effects, phagocytosis, and cytokine production, with an important role in autoimmune disease species in particular (Wang et al., 2021). In recent years about the role of Ficolin in the development of various tumorigenic species has also been confirmed. However, there is no clear report about its immune mechanism and immunotherapy with tumors. We found that the Ficolin family was associated with immune cell infiltration in HCC by analysis, especially FCN1 and FCN3, and their expression was significantly correlated with numerous immune cells. Also, the results of this study showed that FCNs showed significant correlation with numerous immune functions, and FCN1 and FCN3 were more closely associated with immune functions. The results of immune escape analysis showed that the expression of FCN1 and FCN3 were positively correlated with the risk of immune escape, so we can infer that there is a link between FCN1 and FCN3 and the immunotherapeutic effect of HCC. By analyzing the immune mechanism and immunotherapy of FCNs and HCC, we can infer that FCNs can be used as a new immunotherapeutic target for HCC, and their specific mechanism of action deserves our in-depth study, where we speculate whether Ficolin/complement system/tumor immunotherapy can be used as a pathway for immunotherapy of HCC.

Analysis of the results from the present study revealed a higher diagnostic and prognostic value of FCN3, and a previous transcriptomic and genomic analysis study showed that FCN3 showed a consistent reduction in expression in HCC and hepatoblastoma (HPBL) compared to normal liver tissue, consistent with the results validated in the present study (Luo et al., 2006). It was also found that HCC patients showed significantly higher expression of FCN3 in serum after radiofrequency ablation, and this study suggests that FCN3 may be a biomarker for the therapeutic efficacy of radiofrequency ablation and a potential target for immunotherapy of HCC (Shen et al., 2018). However, previous studies have also shown that FCN2 and MBL can be used as biomarkers for the progression of chronic HCV infection to hepatocellular carcinoma, especially when the conversion from HCV to HCC is followed by a significant increase in FCN2 expression (Jalal et al., 2019). However, FCN2 activity in its findings only started to increase significantly in HCV patients approaching the diagnosis of HCC (1 year before the diagnosis of HCC). Undeniably, the results of this study confirmed the diagnostic value of FCN2 in HCC. However, it did not seem to show higher value in the early diagnosis of HCC.

Although the combination of previous studies and our current analysis suggests that FCNs have some association with the development of HCC and immunotherapy, more experiments are needed to confirm and analyze their specific mechanisms of action, thus facilitating the clinical application of FCNs as prognostic indicators or immunotherapeutic targets for HCC.

## Conclusion

In summary, our study provides new insights into the link between the Ficolin family and immunotherapy in HCC, where FCN3 presents a higher research value as a possible prognostic biomarker and immunotherapeutic target for HCC.

## Data Availability

Publicly available datasets were analyzed in this study. This data can be found here: http://timer.cistrome.org/ https://gepia.cancer-pku.cn http://ualcan.path.uab.edu/ https://kmplot.com/analysis/ https://www.tcgaportal.org https://www.cbioportal.org https://portal.gdc.cancer.gov/.
