# Supplementary figures and images for: Comprehensive Analysis of Prognostic Value and Immune Infiltration of Ficolin Family Members in Hepatocellular Carcinoma
Source: Front Genet. 2022 Jul 19;13:913398. doi: 10.3389/fgene.2022.913398 (PMC9343789; doi:10.3389/fgene.2022.913398)

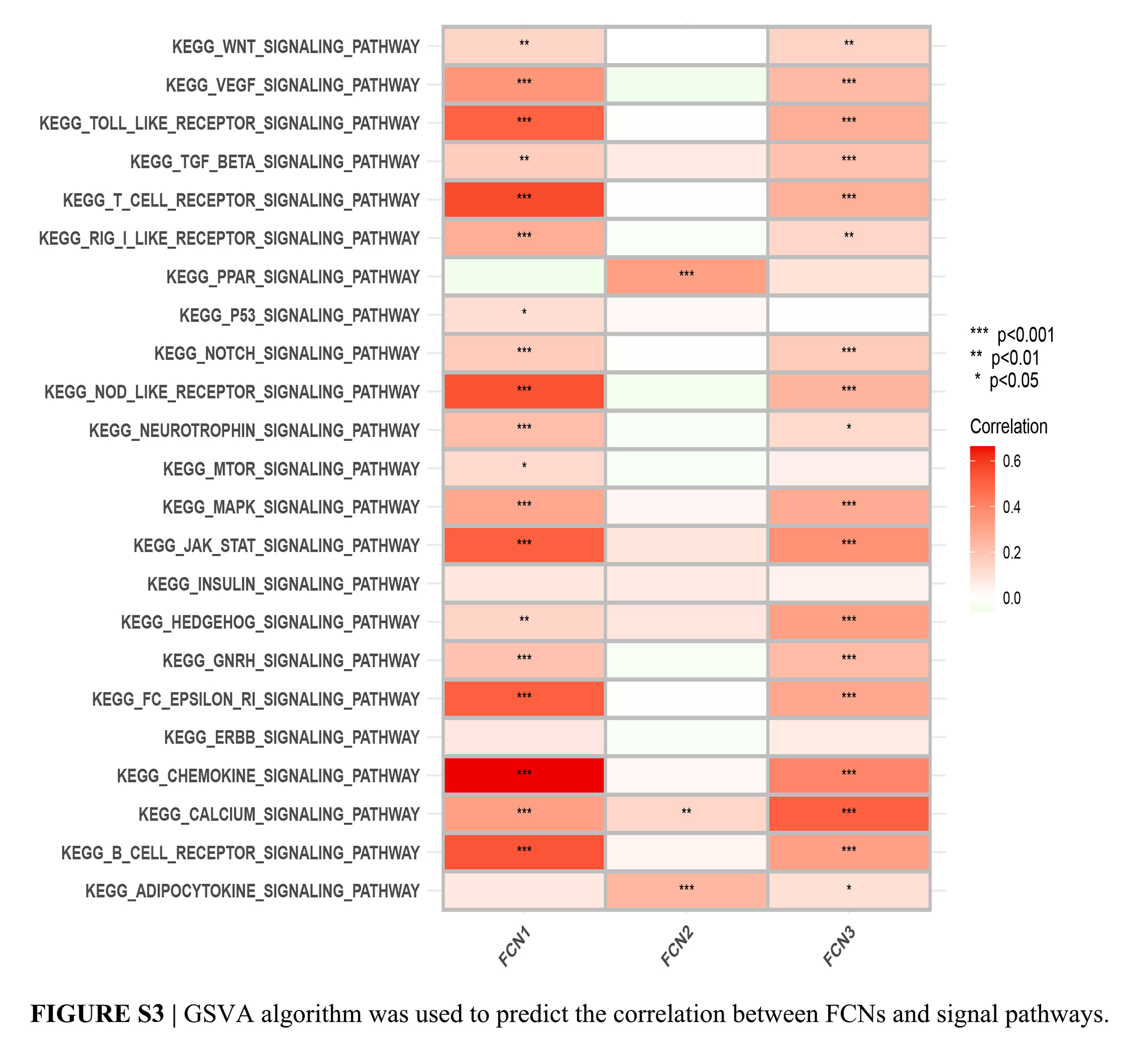

Supplement: Supplementary file 1 [file Image3.JPEG]

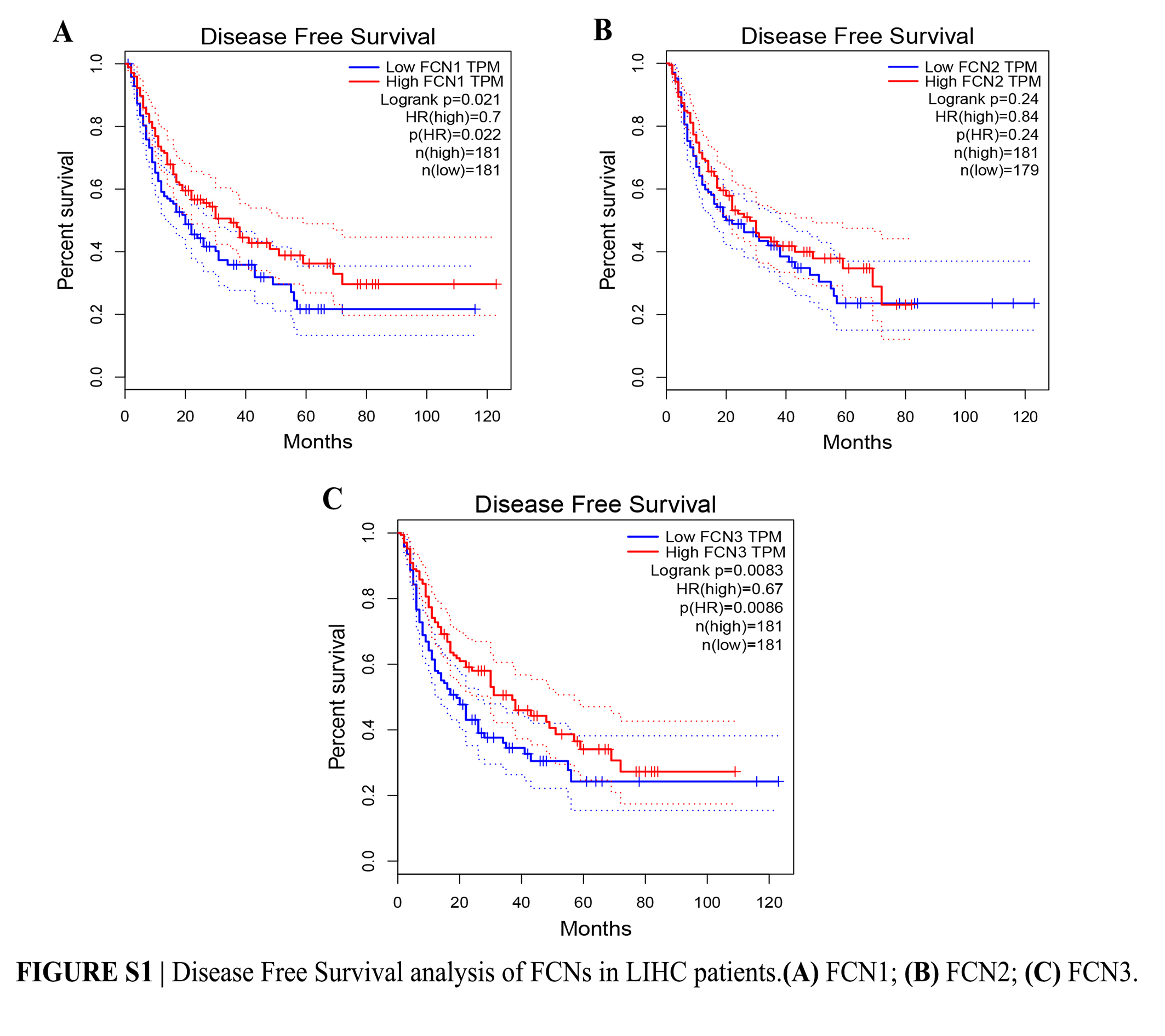

Supplement: Supplementary file 3 [file Image1.JPEG]

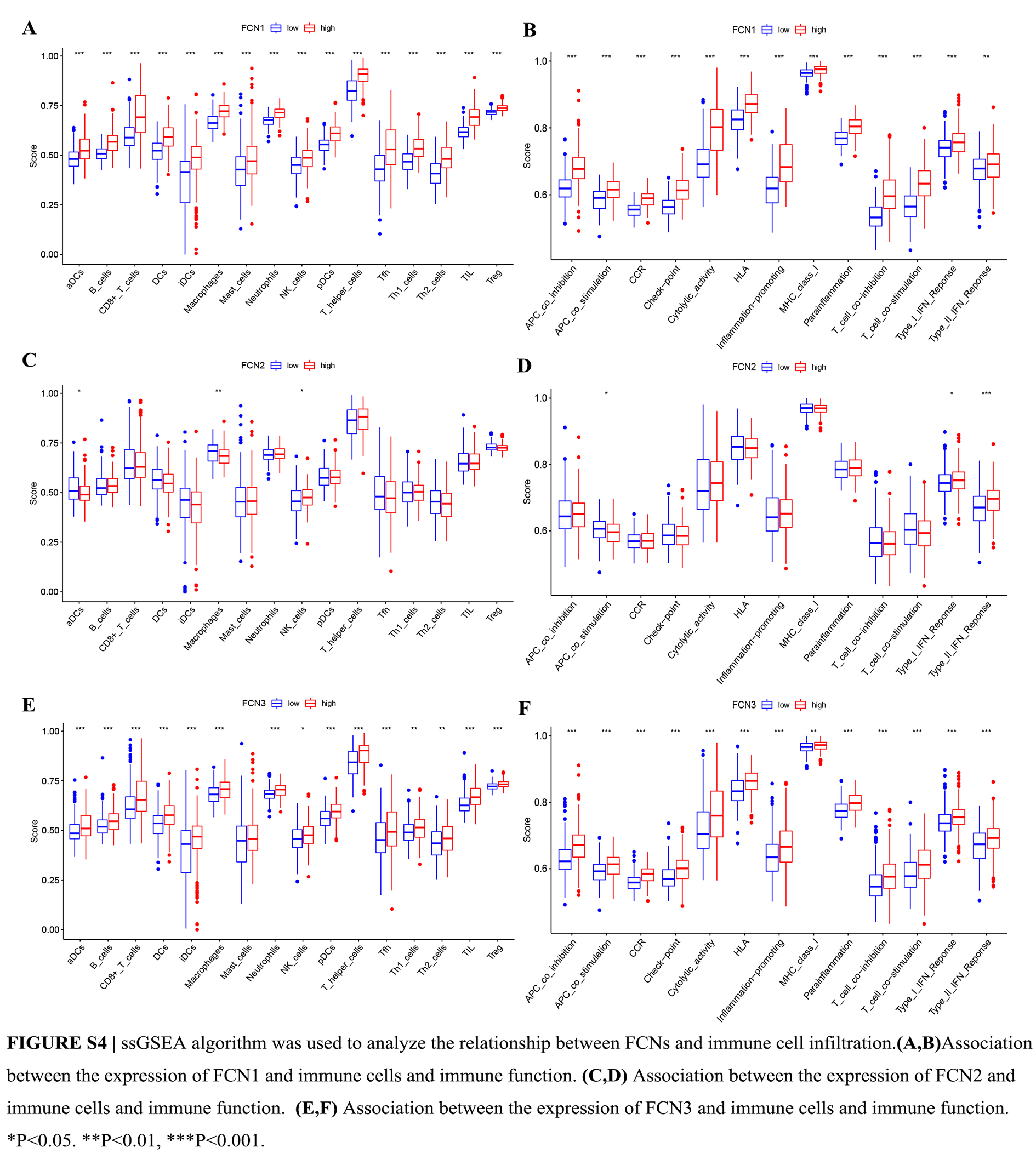

Supplement: Supplementary file 4 [file Image4.JPEG]

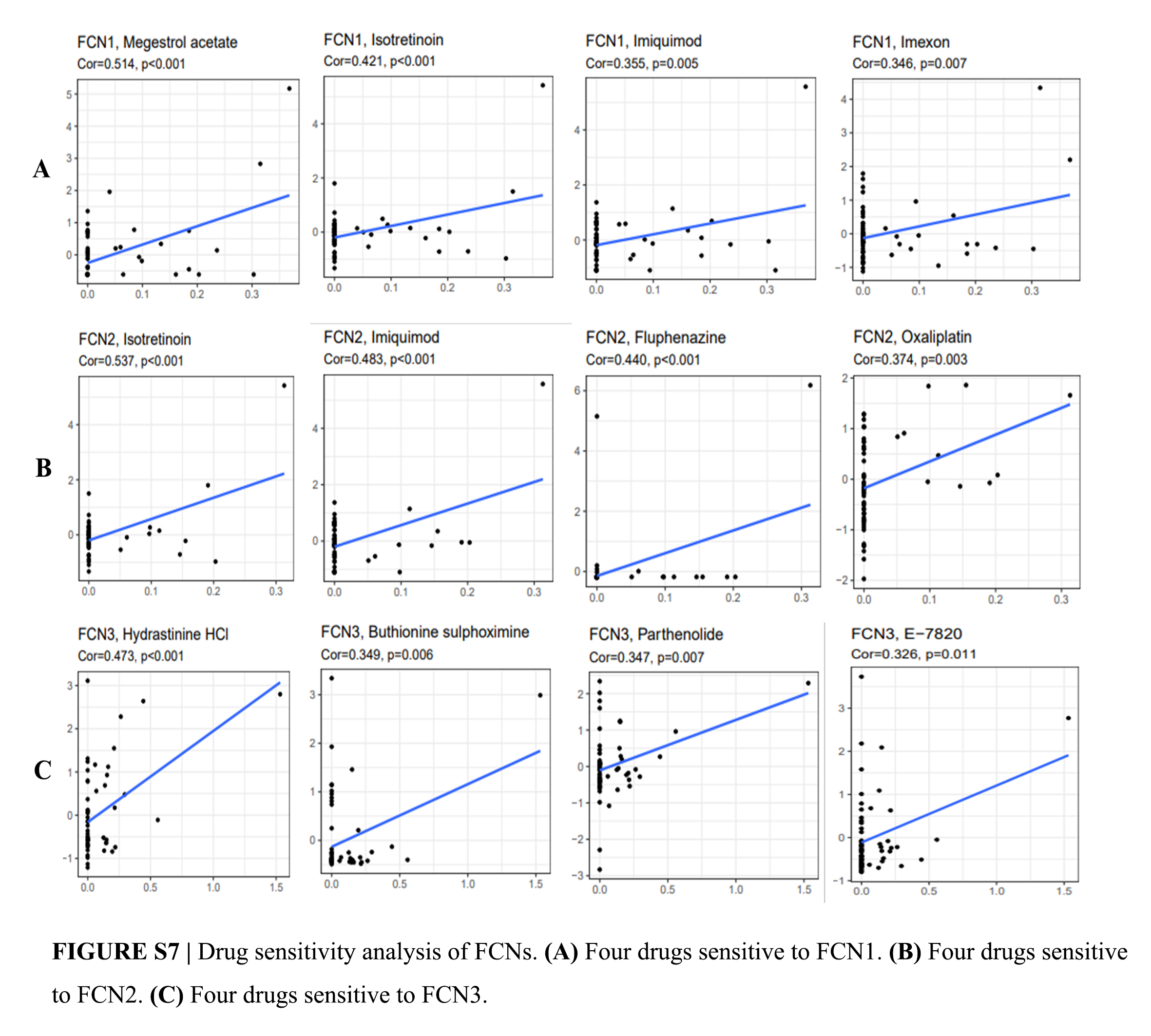

Supplement: Supplementary file 5 [file Image7.JPEG]

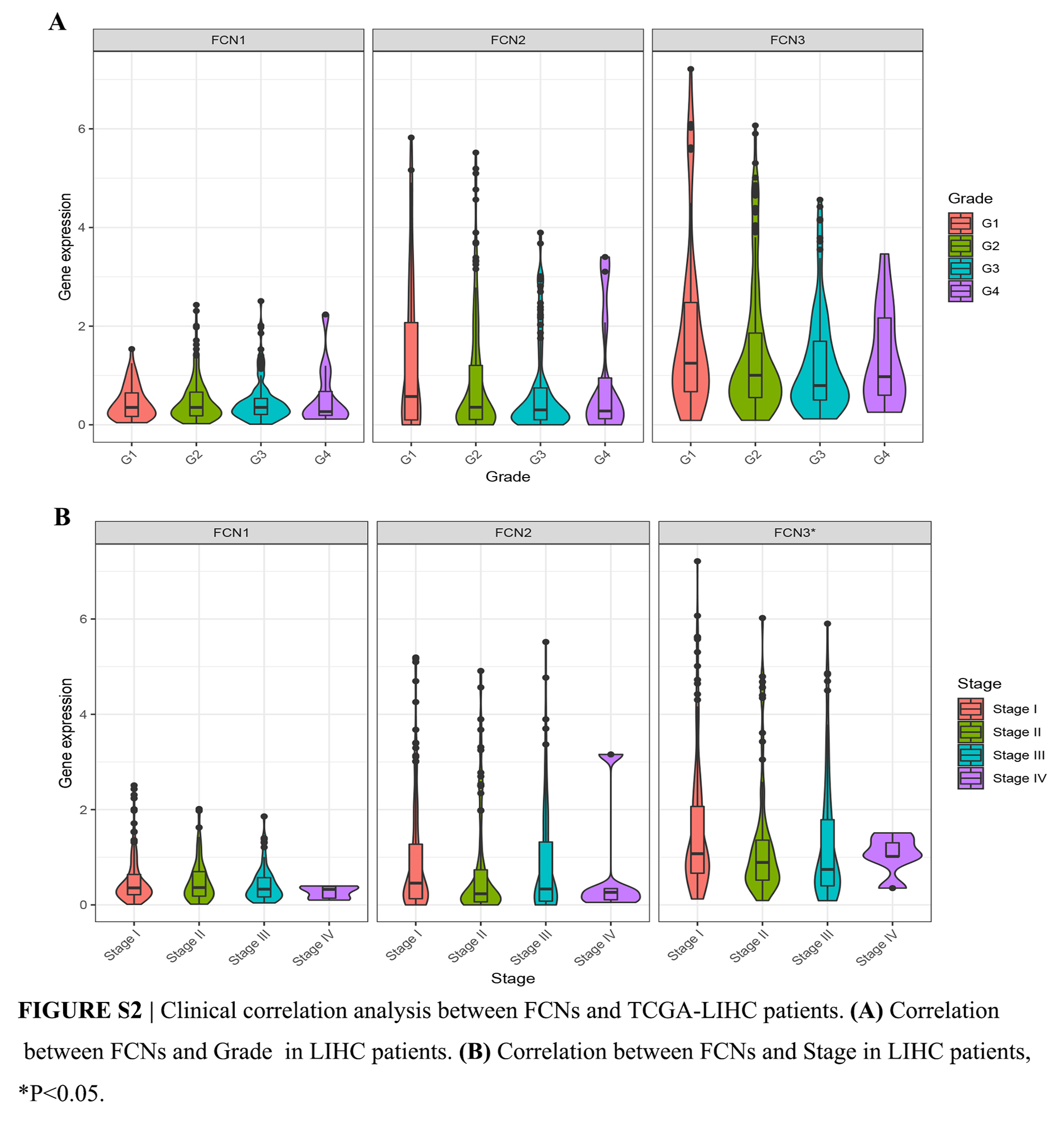

Supplement: Supplementary file 6 [file Image2.JPEG]

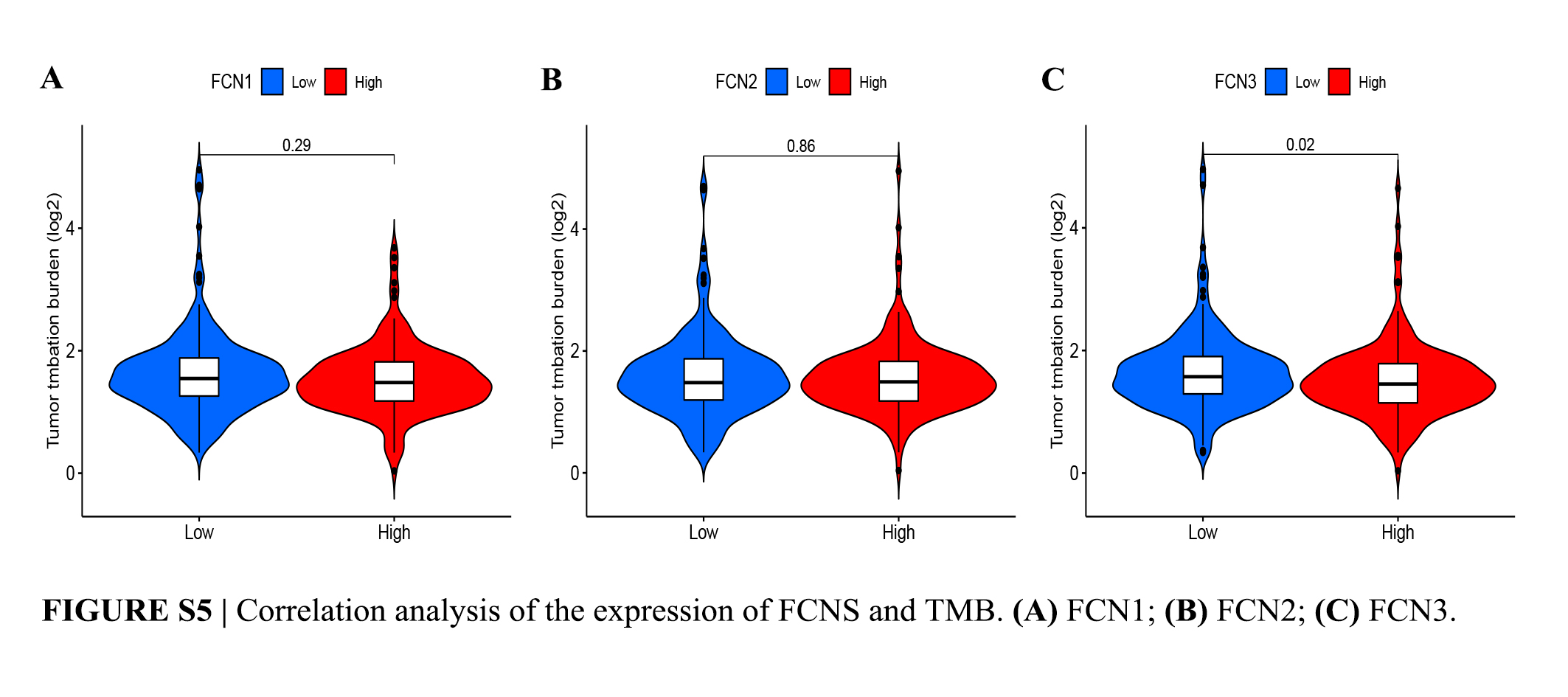

Supplement: Supplementary file 7 [file Image5.JPEG]

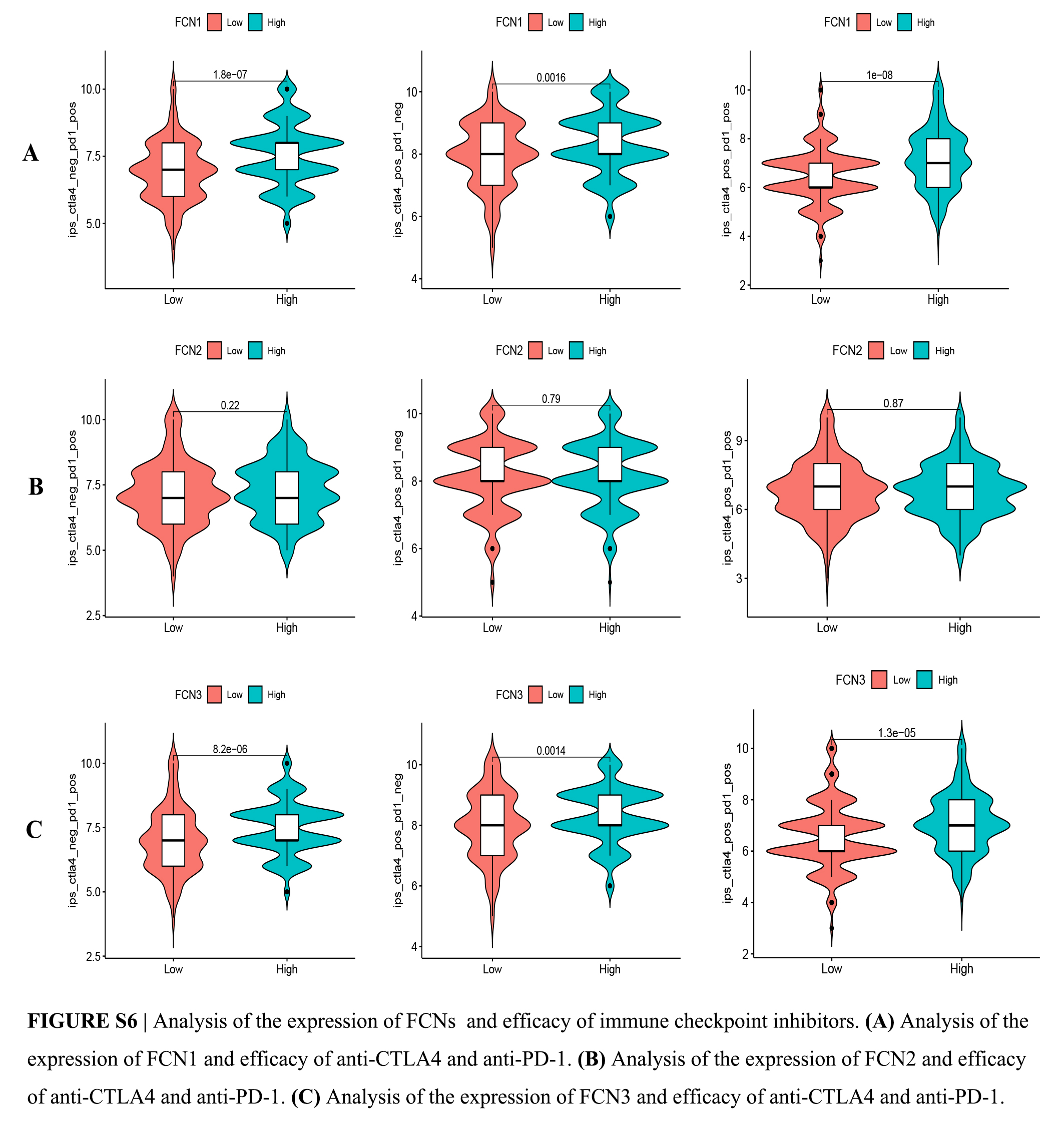

Supplement: Supplementary file 8 [file Image6.JPEG]
