# Supplementary material for: Comprehensive Analysis of Prognostic Value and Immune Infiltration of Ficolin Family Members in Hepatocellular Carcinoma
Source: Front Genet. 2022 Jul 19;13:913398. doi: 10.3389/fgene.2022.913398 (PMC9343789; doi:10.3389/fgene.2022.913398)
Supplement: Supplementary file 2 [file Table1.DOCX]

|  | **FCN1** | |  | **FCN2** | |  | **FCN3** | |
| --- | --- | --- | --- | --- | --- | --- | --- | --- |
| **Immune cells** | **r** | **p** | **Immune cells** | **r** | **p** | **Immune cells** | **r** | **p** |
| aDC | 0.592 | <0.001 | CD8 T cells | 0.158 | 0.002 | aDC | 0.142 | 0.006 |
| B cells | 0.620 | <0.001 | Cytotoxic cells | 0.160 | 0.002 | B cells | 0.357 | <0.001 |
| CD8 T cells | 0.443 | <0.001 | DC | 0.161 | 0.002 | CD8 T cells | 0.279 | <0.001 |
| Cytotoxic cells | 0.604 | <0.001 | Eosinophils | 0.199 | <0.001 | Cytotoxic cells | 0.341 | <0.001 |
| DC | 0.520 | <0.001 | Neutrophils | 0.228 | <0.001 | DC | 0.383 | <0.001 |
| Eosinophils | 0.257 | <0.001 | NK cells | 0.165 | 0.001 | iDC | 0.343 | <0.001 |
| iDC | 0.625 | <0.001 | Tcm | 0.163 | 0.002 | Macrophages | 0.382 | <0.001 |
| Macrophages | 0.560 | <0.001 | TFH | -0.136 | 0.008 | Mast cells | 0.310 | <0.001 |
| Mast cells | 0.319 | <0.001 | Th2 cells | -0.180 | <0.001 | Neutrophils | 0.383 | <0.001 |
| Neutrophils | 0.526 | <0.001 |  |  |  | NK CD56dim cells | 0.272 | <0.001 |
| NK CD56bright cells | 0.238 | <0.001 |  |  |  | NK cells | 0.508 | <0.001 |
| NK CD56dim cells | 0.421 | <0.001 |  |  |  | pDC | 0.284 | <0.001 |
| NK cells | 0.235 | <0.001 |  |  |  | T cells | 0.316 | <0.001 |
| pDC | 0.184 | <0.001 |  |  |  | Tem | 0.369 | <0.001 |
| T cells T helper cells | 0.448 | <0.001 |  |  |  | TFH | 0.229 | <0.001 |
| Tem | 0.337 | <0.001 |  |  |  | Tgd | 0.315 | <0.001 |
| TFH | 0.457 | <0.001 |  |  |  | Th1 cells | 0.306 | <0.001 |
| Tgd | 0.232 | <0.001 |  |  |  |  |  |  |
| Th1 cells | 0.645 | <0.001 |  |  |  |  |  |  |
| Th2 cells | 0.236 | <0.001 |  |  |  |  |  |  |
| TReg | 0.326 | <0.001 |  |  |  |  |  |  |

**Table S1 |** Results of correlation between FCNs and immune cells infiltration.
